# Small RNA sequencing identifies serum tDR-1:34-Gly-GCC tiRNA levels as a biomarker for survival in amyotrophic lateral sclerosis

**DOI:** 10.1016/j.isci.2026.115636

**Published:** 2026-04-07

**Authors:** Koen C. Demaegd, Lindy Kool, Sharada Baindoor, Paul D. Donovan, Junyi Su, Henk-Jan Westeneng, Ruben P.A. van Eijk, Grainne Geoghegan, Elena Perez Morrissey, Luise Halang, Koen Poesen, Pegah Masrori, Hesham A.Y. Gibriel, Elisabeth Jirström, Morten T. Venø, Jørgen Kjems, Orla Hardiman, Jan H. Veldink, Kevin Kenna, Leonard H. van den Berg, Philip Van Damme, Jochen H.M. Prehn, Michael A. van Es

**Affiliations:** 1 Department of Neurology, UMC Utrecht Brain Center, University Medical Center Utrecht, Utrecht University, Utrecht, the Netherlands; 2 Department of Translational Neuroscience, UMC Utrecht Brain Center, University Medical Center Utrecht, Utrecht University, Utrecht, the Netherlands; 3 Department of Physiology and Medical Physics, RCSI Centre for Systems Medicine, Royal College of Surgeons in Ireland, St Stephen’s Green, Dublin 2, Ireland; 4 Department of Molecular Biology and Genetics, Interdisciplinary Nanoscience Center (iNANO), Aarhus University, Aarhus C, Denmark; 5 Omiics ApS, Aarhus N, Denmark; 6 FutureNeuro Research Ireland Centre, Royal College of Surgeons in Ireland, Dublin 2, Ireland; 7 Laboratory for Molecular Neurobiomarker Research, KU Leuven, Leuven, Belgium; 8 Laboratory of Neurobiology, Neuroscience Department, KU Leuven and Neurology Department, University Hospitals, Leuven, Belgium; 9 Institute of Neuroscience, UCLouvain, Brussels, Belgium; 10 Department of Neurology, Cliniques Universitaires Saint-Luc, Brussels, Belgium; 11 Academic Neurology Unit, Trinity Biomedical Sciences Institute, Trinity College Dublin, Dublin, Ireland

**Keywords:** nucleic acids, molecular neuroscience, sequence analysis

## Abstract

Survival is highly variable in amyotrophic lateral sclerosis (ALS), complicating prognosis and clinical trial design. Despite advances in biomarker development, accessible prognostic tools are limited. Small non-coding (snc) RNAs are a recently discovered biomarker class showing differential regulation across neurodegenerative diseases, including ALS. Here, we explored changes in sncRNAs over time in ALS. We performed small RNA sequencing in a discovery cohort of 116 longitudinal serum samples from ALS 40 patients collected at 3- to 4-month intervals and identified tRNA-derived stress-induced RNA (tiRNA) tDR-1:34-Gly-GCC as the top sncRNA to increase over time. The finding was validated using TaqMan PCR and replicated in an independent cohort of 35 patients. Both univariate and joint model analyses showed that higher tDR-1:34-Gly-GCC levels correlated with shorter survival. Given that the translation of mRNAs and stress-induced translation inhibition are dysregulated in ALS and linked to familial ALS genes, combined with these findings, serum tDR-1:34-Gly-GCC tiRNA levels hold potential as a prognostic biomarker and outcome measure in clinical trials.

## Highlights

• tDR-1:34-Gly-GCC tiRNA increases along the disease course in ALS
• Higher tDR-1:34-Gly-GCC tiRNA levels are correlated with shorter survival
• tDR-1:34-Gly-GCC tiRNA is a promising, pathophysiology-driven prognostic biomarker

## Graphical abstract

**Figure:**
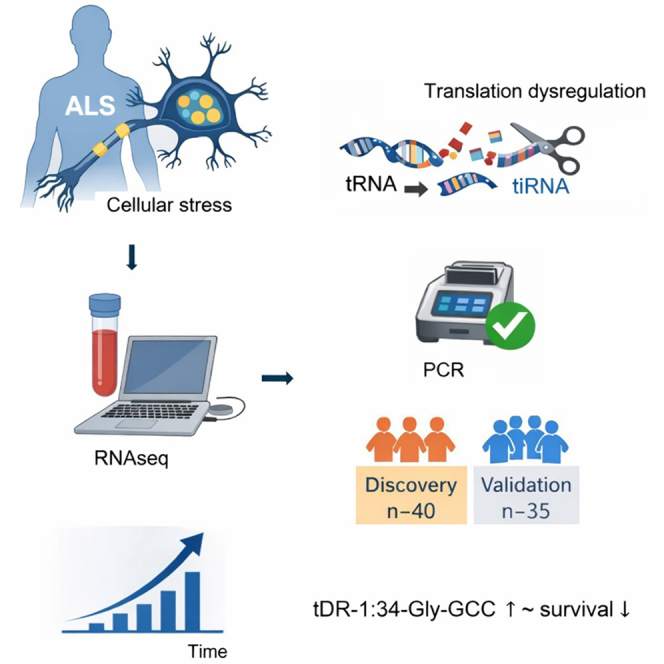

## Introduction

Amyotrophic lateral sclerosis (ALS) is a progressive disorder characterized predominantly by the degeneration of upper and lower motor neurons, resulting in progressive weakness and spasticity. It typically affects people between 40 and 75 years. The average survival is 3–4 years after onset,^1^ but it ranges from several months to over 10 years in rare cases. Onset is mostly manifested by weakness in the limbs (spinal onset), 20–30% of patients manifest with bulbar dysfunction (e.g., difficulty swallowing, altered speech, or tongue weakness). In terms of clinical manifestations, genetics, and pathophysiology, ALS is highly heterogeneous^2^^,^^3^ and partially overlaps with frontotemporal dementia.^4^

The wide range in survival makes it challenging to provide patients with an accurate prognosis. It also complicates conducting clinical trials, as it is crucial that trial outcomes are attributable to the effect of the investigational product and are not driven by differences in the underlying patient characteristics between study arms. With the objective of providing more accurate prognoses and the enrollment of homogeneous patient populations in clinical studies, prognostic models have been developed using clinical characteristics, such as the ENCALS Survival Prediction Model and risk profile.^5^^,^^6^ Similarly, “wet” biomarkers have been explored, from which neurofilament light chain (NfL) has emerged as the leading candidate to aid ALS therapy development. Studies have shown that it holds value as a prognostic biomarker when measured early in the disease and can be used for stratification and/or randomization. Furthermore, reductions in NfL are potentially indicative of efficacy and can therefore be used to prioritize compounds in the early stages of development.^7^ Despite these major advances, our understanding of phenotypic variability in ALS remains incomplete, and there remains an urgent need for additional biomarkers.

Small non-coding (snc) RNAs form a relatively novel class of biomarkers that due to their stability in biological fluids have potential across multiple neurodegenerative diseases. MicroRNAs (miRNAs) are the most studied sncRNA compared to other classes, given their diverse roles in gene regulation.^8^ For instance, in ALS, circulating microRNA miR-181 is a prognostic biomarker with a similar performance to NfL, which, when combined, leads to superior prognostication capacity.^9^^,^^10^

Here we utilized an unbiased approach to detect changes in different types of sncRNAs over time in ALS through a small RNA-seq study and subsequent bioinformatic analysis of differentially expressed sncRNAs. We obtained data from a discovery cohort of 40 patients with ALS that provided serum samples at 3 to 4 months intervals, similar to a visit schedule in a clinical trial. Small RNA-seq results were validated by qPCR, replicated in an independent cohort of 35 patients, and tested for association with survival. This led to the identification of 5′GlyGCC tRNA-derived stress-induced RNA (tiRNA) (5′GlyGCC tiRNA/tDR-1:34-Gly-GCC) as a potential biomarker for survival in ALS.

## Results

### Baseline characteristics of the study participants

Descriptive characteristics are shown in Table 1. Comparing our cohorts, the discovery cohort is on average slightly younger (*p* = 0.006) and contains not significantly more *C9orf72* carriers. Variability in ALSFRS-R slope is larger, while the bulbar region of onset, survival and other parameters are comparable. None of the genetically tested patients carried an *ANG* mutation. Changes in values over time within individual patients were the primary focus of this study, and as such, the difference in age between the cohorts was not considered a relevant factor for analysis.

**Table 1:** Clinical characteristics

	Discovery (*N* = 40)	Replication (*N* = 35)
Male sex, n (%)	26 (65%)	26 (74%)
FTD|ALS-CI (n)	1|0	2|1
Age of onset (yrs.)^a^	55 [46–63]	61 [55–69]
Bulbar onset, n (%)	10 (25%)	11 (31%)
Diagnostic delay (mo.)	9 [6–12]	9[6–14]
Disease duration at inclusion (mo.)	12 [7–17]	9 [7–17]
Survival since onset (yrs.)	3.6 [2.2–5.1]	4.2 [2.2–7.4]
Survival since inclusion (yrs.)	2.0 [1.5–4.0]	1.4 [0.8–2.7]
ALSFRS-R slope/month	0.4 [0.3–0.7]	0.7 [0.3–1.2]
C9orf72 carriers, n (%)	4 (10%)	0 (0%)

Median [IQR]; n (%).

a statistical difference between the two groups.

### RNA seq – Selection of tDR-1:34-Gly-GCC as potential biomarker

**Figure 1:**
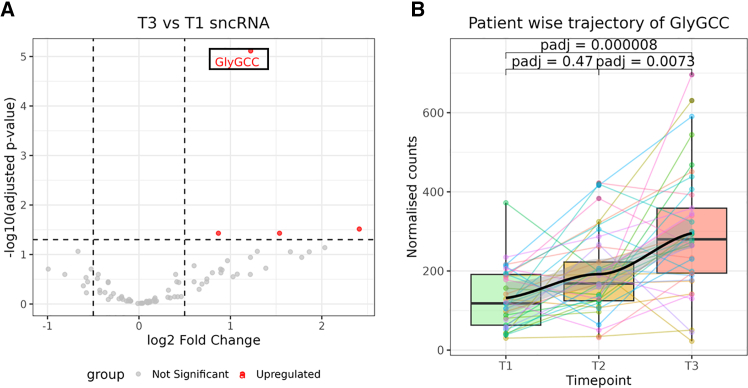
Expression of tDR-1:34-Gly-GCC tiRNA from small RNA-seq data (A) Volcano plot shows the results of T3 versus T1 small RNA seq data, where tDR-1:34-Gly-GCC (enclosed within the black box) was selected as most promising tsRNA longitudinal biomarker. tDR-1:34-Gly-GCC log2 fold change: 1.22, *p*-value: 9.88×10^−8^ and adjusted *p*-value 7.7 x 10^−6^ (*n* = 37 at T1 and *n* = 40 at T3). (B) Bar plots show the expression of tDR-1:34-Gly-GCC at timepoints T1 (*n* = 33), T2 (*n* = 37) and T3 (*n* = 37) with LOESS overlay. Outliers were removed at each timepoint using Tukey’s method. Each individual point and line across timepoints represents a patient. x axis represents the timepoints and y axis has DESeq2 normalized counts. Statistical significance between timepoints represents the adjusted *p*-value obtained using DESeq2. Boxplots are plotted to represent the median and interquartile range.

Differential expression analysis showed a significant longitudinal increase of several small nucleolar RNAs (SNORD) and tiRNAs, from which tDR-1:34-Gly-GCC tiRNA (Figure S2) was selected as most promising based on statistical significance and quantity of expression (Figure 1A). tiRNAs are generated through cleavage of transfer RNAs by angiogenin protein in their anticodon loop, generating 30-40-nucleotide-long tiRNAs.^11^^,^^12^

### Longitudinal increase of tDR-1:34-Gly-GCC validated by qPCR

**Figure 2:**
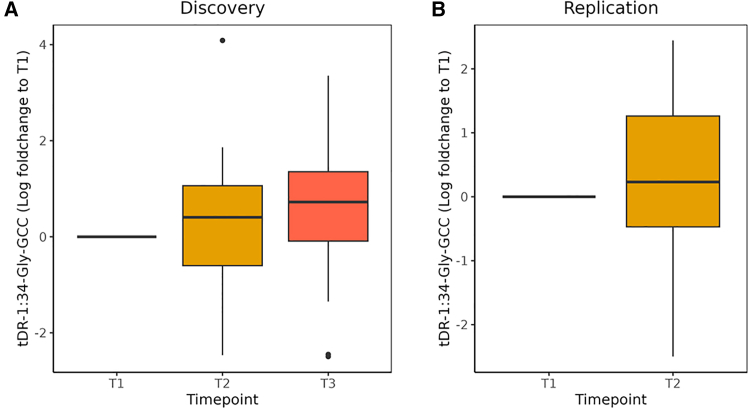
Serum tDR-1:34-Gly-GCC increases over time in patients with ALS Boxplots of serum tDR-1:34-Gly-GCC expression measured by TaqMan assay in A. Discovery cohort at timepoint T1 (*n* = 36), T2 (*n* = 36) and T3 (*n* = 36). (B) Replication cohort at T1 (*n* = 24) and T2 (*n* = 24). x axis indicates the timepoints, and y axis represents log2 fold change values of tDR-1:34-Gly-GCC levels relative to T1. Boxplots are plotted to represent the median and interquartile range. Horizontal gray line indicates timepoint 1 (0 months). A linear mixed-effects model was conducted. Serum tDR-1:34-Gly-GCC is significantly higher at timepoints 2 (3–4 months) and 3 (6 to 8 months) compared to timepoint 1.

Next, we validated our small RNA seq findings using TaqMan-based PCR analysis of tDR-1:34-Gly-GCC levels as an assay that can also be performed in routine biochemistry laboratories. Intra-assay variation was minimal with coefficient of variation (CV) values ranging approximately 3%, and inter-assay variation showed CV values ranging from 7 to 9%. The lowest concentration measured was well above the lower limit of quantification. In both the discovery and replication cohorts, we detected a longitudinal increase in tDR-1:34-Gly-GCC levels during disease progression with each additional year associated with an average increase in Fold Change of 0.5 in the discovery cohort (b = 0.5, *p* = 0.009) and 0.9 in the replication cohort (b = 0.9, *p* = 0.01), as shown in Figure 2. It is important to note that, because fold change was calculated using the 2ˆ–ΔΔCt method, a positive estimate for Time reflects an increase in tDR-1:34-Gly-GCC expression over time, even if the absolute Fold Change values are less than 1. Of note, higher tDR-1:34-Gly-GCC levels correlated with shorter survival in a joint model (HR: 1.65; 95% CI: 1.05–5.02). The longitudinal increase of tDR-1:34-Gly-GCC was independent of gender, site, and age of onset. While tDR-1:34-Gly-GCC levels were a predictor of survival, we could not find a correlation with progression, as expressed through decline in ALSFRS-R score (estimate = 0.003, *p* = 0.93) or ALSFRS-R slope (estimate = −0.066, *p* = 0.81), as assessed using linear mixed models.

### Angiogenin increases longitudinally

tiRNAs are generated through cleavage of transfer RNAs by the ribonuclease angiogenin under circumstances of cellular stress.^12^^,^^13^

**Figure 3:**
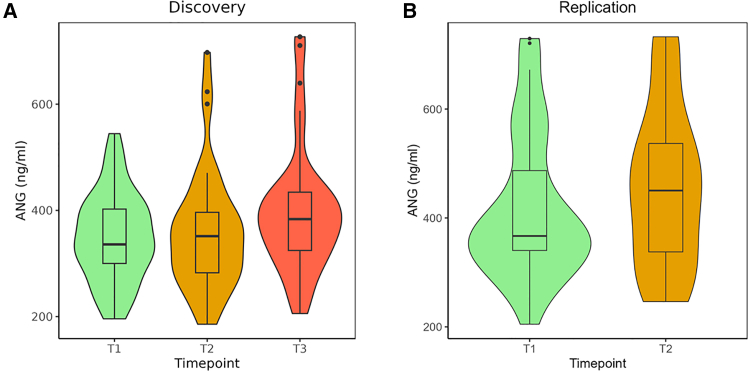
Serum ANG increases over time in patients with ALS Violin plots show ANG levels in serum measured using ELISA. (A) ANG (ng/mL) levels in the discovery cohort at timepoint T1 (*n* = 38), T2 (*n* = 38), T3 (*n* = 40). (B) ANG (ng/mL) levels in the replication cohort at timepoint T1 (*n* = 33) and T2 (*n* = 35). X axis indicates the timepoints, and y axis shows the ANG measured in ng/ml. Boxplots are plotted to represent the median and interquartile range.

We therefore next explored whether angiogenin levels also increased during disease progression in our largely sporadic ALS cohort. We noted a modest but statistically significant increase in angiogenin serum protein levels over time (b = 0.038, *p* = 0.013) (Figure 3). However, there was no correlation between angiogenin and tDR-1:34-Gly-GCC. No significant correlation was found with gender, age of onset, site of onset, mutation status, survival, or progression rate. These findings suggest that tDR-1:34-Gly-GCC levels in serum as a readout of angiogenin activity have more prognostic value than angiogenin serum levels themselves.

### Neurofilament light levels

We finally wanted to explore whether tDR-1:34-Gly-GCC also correlated with levels of NfL, currently the only widely accepted biomarker. With CV values of 3.9% for intra-assay variation and 2.4% for inter-assay variation, both are minimal. Since NfL was not normally distributed, we applied a log transformation (log(NfL)) to approximate normality. NfL correlated with shorter survival in a univariate model (HR = 1.005, 95% CI: 1.003–1.007; *p* < 0.001). NfL showed a slight increase over time (b = 0.053, *p* = 0.024) and when correcting for gender, site and age of onset in a multivariate model, showed a correlation with the ALSFRS-R slope (b = 0.36, *p* = 0.02). NfL did not correlate with tDR-1:34-Gly-GCC levels. As such, NfL and tDR-1:34-Gly-GCC are independent predictors of survival, likely due to being biomarkers for different biological processes (neurodegeneration vs. cellular stress).

## Discussion

In this study, we show that serum levels of tDR-1:34-Gly-GCC increase over time in patients with ALS, and identify tDR-1:34-Gly-GCC as a potential biomarker for survival, thereby opening up the field of tiRNAs as biomarkers of disease progression in ALS. Of note, tDR-1:34-Gly-GCC levels correlated inversely with survival. Aberrant stress signaling and control of protein translation are critical events in ALS. Hence, tiRNAs formed via stress-induced cleavage of transfer RNAs could be a biological readout of a stress response in ALS.^14^ We hypothesize that there is more neuronal cell stress in cases with a shorter survival, hence explaining the presence of higher tDR-1:34-Gly-GCC levels in these patients. While the increment of tDR-1:34-Gly-GCC over time held strong in subgroups based on region of disease onset, there was no significant difference in tDR-1:34-Gly-GCC levels between bulbar and spinal onset.

The formation of these tiRNAs, such as tDR-1:34-Gly-GCC, relies on the ribonucleolytic activity of ANG.^15^ Of note, our findings of elevated tDR-1:34-Gly-GCC levels across our cohort also point to the role of angiogenin in sporadic, bulbar, and spinal onset ALS. Further, we show that serum ANG levels also increase over time in patients with ALS. Both increased angiogenin activity as well as increased angiogenin levels may cause the increase in tDR-1:34-Gly-GCC levels over time. The dissociation between angiogenin protein levels and tDR-1:34-Gly-GCC increase could hence be caused by an increase in angiogenin activity, but alternative nucleases (e.g., RNase A) might play a role as well. tiRNAs such as tDR-1:34-Gly-GCC are predicted to form stable hairpin-like structures that may be protected from degradation. It is therefore also possible that the stronger increase in tDR-1:34-Gly-GCC levels compared to angiogenin protein levels is a consequence of different degradation rates. This, coupled with their ease of detection by PCR, may make tDR-1:34-Gly-GCC an interesting “wet” biomarker of survival.

ALS pathophysiology is considered a multistep process whereby neuronal stress and neuronal damage accumulate over many years.^16^ The different stresses that have been linked to the development of ALS include excitotoxicity, proteasomal stress due to intracellular protein aggregates, endoplasmic reticulum stress, defects in autophagy, mitochondrial dysfunction, and oxidative stress. Neurons affected by these processes can respond with the formation of “stress granules,” as seen in ALS and FTD.^17^ These stress granules and other structures, such as “P bodies,” are endogenous mRNA-protein granules that form during cellular stress, in which mRNAs may be stored, and protein translation is repressed.^17^^,^^18^ Many protein products of genes linked to familial forms of ALS and FTD, including TARBDP/TDP-43, FUS, and angiogenin, co-localize to stress granules, highlighting the importance of this stress response in disease onset and progression.^18^^,^^19^ Stress granules also contain tRNAs, 5.8S ribosomal RNA (rRNA), and sncRNA.^17^ tiRNAs have been suggested to play a crucial role in the regulation and resolution of cellular stress responses.^13^ tDR-1:34-Gly-GCC in particular has been shown to be elevated under oxidative, proteasomal, and excitotoxic stress in mouse primary cortical neurons as well as the spinal cord of TDP-43^A315T^ mice compared to Wildtype.^20^ Loss-of-function mutations in the *ANG* gene, which encodes the angiogenin protein, have also been identified in patients with ALS (27, 28). Additionally, tiRNAs containing high guanine content can form G-quadruplexes, which have demonstrated neuroprotective activity against excitotoxicity, proteotoxicity, and serum starvation in human motor neurons.^14^ Recent work from our and other groups also identified other tiRNAs to be generated in neural cells by ANG cleavage and to be elevated in mouse models of ALS and in ALS patient serum samples when compared to controls.^21^^,^^22^

### Limitations of the study

Being an exploratory study, this research has a few limitations. Firstly, given the relevance of 5′tiRNAs in ALS-relevant stress conditions, diagnosis and progression^20^^,^^21^^,^^22^^,^^23^^,^^24^^,^^25^ the RNA-seq procedure enabled us to detect 5′ tsRNAs and identify tDR-1:34-Gly-GCC as a marker of survival. However, due to the RNA extraction method involved prior to sequencing, we acknowledge that not all sncRNAs may have been detected. Conversely, we cannot fully exclude the possibility that our TaqMan assay, which was designed to detect the longer tiRNA fragment, may also have captured shorter 5′tiRNA fragments generated by ANG. For our validation cohort, we only had the availability of samples from two timepoints, unlike the three timepoints in the discovery cohort. Further, both cohorts had a modest size. While in another paper by our group, tDR-1:34-Gly-GCC did not show to be upregulated in patients with ALS compared to healthy controls, we have no samples from healthy controls collected over 6–8 months, fitting into the longitudinal study design, which could clarify this finding.^23^ Additionally, our study excluded patients with ALS whose survival time was shorter than six months post-diagnosis, as our methodology required data from at least two timepoints following an ALS diagnosis. This could affect the strength of the association between the biomarker and survival. The source of tDR-1:34-Gly-GCC is also still unclear. As we hypothesize that tDR-1:34-Gly-GCC is released due to neuronal stress, it would be interesting to perform measurements in cerebrospinal fluid. However, another possible source was recently suggested by Shen et al.,^26^ showing that 5′tiRNA-Gly was up-regulated after muscle injury. It is also possible that tiRNAs are generated through inflammatory pathways and/or activated microglia, as demonstrated for several ALS-associated miRNAs.^27^ Finally, an exogenous spike-in was used to control extraction efficiency, which, however, does not correct for biological input variability. As there are no validated endogenous controls for our system that are appropriate for this RNA class (tsRNAs), this method was deemed the most reliable and biologically independent method to oversee technical variability.

Concluding, our study identifies serum tDR-1:34-Gly-GCC as a promising biomarker for ALS survival and a potential outcome measure in clinical trials. Its correlation with shorter survival suggests it reflects neuronal stress. The stability and detectability of tiRNAs make them attractive liquid biomarkers. While our findings support tDR-1:34-Gly-GCC as a prognostic marker, further validation in larger cohorts is needed.

## Resource availability

### Lead contact

Requests for further information and resources should be directed to and will be fulfilled by the lead contact: Prof Jochen HM Prehn (jprehn@rcsi.ie).

### Materials availability

This study did not generate new unique reagents.

### Data and code availability

Data: The datasets used and analyzed during the current study are available from the repository: Demaegd, K. 2026. “Small RNA Sequencing Identifies Serum tDR-1:34-Gly-GCC tiRNA Levels as a Biomarker for Survival in Amyotrophic Lateral Sclerosis.” Harvard Dataverse. https://doi.org/10.7910/DVN/8VAVUF.
Code: The code used to perform the analyzes in the current study is available from the repository: Demaegd, K. 2026. “Small RNA Sequencing Identifies Serum tDR-1:34-Gly-GCC tiRNA Levels as a Biomarker for Survival in Amyotrophic Lateral Sclerosis.” Harvard Dataverse. https://doi.org/10.7910/DVN/8VAVUF.
Additional information: Any additional information required to reanalyze the data reported in this paper is available from the lead contact upon request.

## Acknowledgments

We are grateful for the patients who participated in this study by donating samples; without them, this research would not have been possible.

Funding: JHMP was supported by Research Ireland (17/JPND/3455, 20/SP/8953 and 21/RC/10294_P2 co-funded under the 10.13039/501100008530European Regional Development Fund and by FutureNeuro and Precision-ALS industry partners). S.B. was supported by the Research Ireland CRT in Genomics Data Science under grant no. 18/CRT/6214. E.J. was supported by the RCSI STAR PhD Programme. PVD is supported by the E. von Behring Chair for Neuromuscular and Neurodegenerative Disorders, the ALS Liga België and the 10.13039/501100004040KU Leuven funds “Een Hart voor ALS” and “Laeversfonds voor ALS Onderzoek.” LHB received grants from the Netherlands ALS Foundation, the 10.13039/501100001826Netherlands Organisation for Health Research and Development (Vici scheme), The European Community's Health Seventh Framework Programme (grant no. 259867; EuroMOTOR), the 10.13039/501100001826Netherlands Organisation for Health Research and Development (STRENGTH project, funded through the 10.13039/100013278EU Joint Programme – Neurodegenerative Disease Research), provides ad hoc consultancy services to Biogen, Ferrer, Amylyx, Takeda, and Argenx (payment received by institution) outside the submitted work, and is the Chair of the European Network for the Cure of ALS (ENCALS) and the Treatment Research Institute for the Cure of ALS (TRICALS). JV has sponsored research agreements with Biogen and AstraZeneca, outside the submitted work. MAE has consulted for Biogen, has received travel grants from Shire (formerly Baxalta), performs work as a medical monitor for an ongoing trial with Ferrer (NCT05178810, fees paid to institution), and receives funding support from the 10.13039/501100001826Netherlands Organisation for Health Research and Development (Vidi scheme), the Thierry Latran Foundation, the Motor Neurone Disease Association, FIGHT-MND, and the ALS Foundation Netherlands. He serves on the board of The Dutch Center for Neuromuscular Diseases (Spierziektencentrum Nederland, SCN) and of EURO-NMD, which is a European Reference Network for the thematic grouping of rare neuromuscular diseases (NMDs). Several authors of this publication are members of the European Reference Network for Rare Neuromuscular Diseases (Euro-NMD). Funding sources had no role in the study design, collection, analysis, or interpretation of data.

## Author contributions

All named authors meet the International Committee of Medical Journal Editors (ICMJE) criteria for authorship for this article and accept responsibility to submit for publication.

J.H.M.P., M.A.E., P.V.D., M.T.V., and J.K. were involved in study design, guiding data acquisition and analysis, as well as participating in writing. K.C.D., L.K., and S.B. executed data verification and analysis, writing, and revisions. P.D., J.S., G.G., E.P.M., L.H., K.P., P.M., H.A.G., E.J., O.H., K.K., and L.H.B. all contributed in different stages of data acquisition. H.J.W., R.P.E., and J.H.V. partake in revising data analysis and writing.

All authors take responsibility for the integrity of the work as a whole. All authors read and approved the final manuscript.

## Declaration of interests

The authors declare no competing interests.

## STAR★Methods

### Key resources table

**Table:** 

REAGENT or RESOURCE	SOURCE	IDENTIFIER
**Antibodies**		

Human ANG	R&D Systems Inc., Minneapolis, MN	DAN00
Neurofilament light	Umandiagnostics AB	10-7001

**Biological samples**		

Serum	Human participants	

**Critical commercial assays**		

miRNeasy Serum/Plasma Kit	Qiagen	Cat no. 217184
QIAseq miRNA Library Kit	Qiagen	Cat. No. 331502
KAPA Library Quantification Kit Illumina Platforms	Kapa Biosystems	KR0405

**Deposited data**		

Dataset		Harvard Dataverse: https://doi.org/10.7910/DVN/8VAVUF
Code used for analysis		Harvard Dataverse: https://doi.org/10.7910/DVN/8VAVUF

**Oligonucleotides**		

custom small RNA Taqman	ThermoFisher Scientific	5’-GCAUUGGUGGUUCAGUGGUAGAAUUCUCGCCUGC-3’

**Software and algorithms**		

Nextflow based pipeline tsRNAsearch	NA	NA
Combat SVA package	Combat	https://doi.org/10.18129/B9.bioc.sva
R version 4.1.2	R	NA

### Experimental model and study participant details

Serum was collected through a population-based study on ALS (Prospective ALS study Netherlands (PAN)) and Leuven Neurobiobank.^28^ All patients were diagnosed ALS according to the Gold Coast criteria.^29^ All participants gave written informed consent according to the Declaration of Helsinki, protocols received approval from the local medical ethical committee (Netherlands NedMec, approval number 05-067/E, Ireland: RCSI REC approval number REC1122bb) and the study complies with the study regulations including GCP.

In our Discovery cohort, 40 patients with ALS had serum collected at diagnosis and 3-4 and 6-8 months after diagnosis. An independent cohort of 35 patients, combined from the Utrecht PAN study and Leuven Neurobiobank (S50354), was selected based on the availability of two consecutive samples 3–4 months apart and used as the Replication cohort.

Descriptive characteristics are shown in Table 1. The age at sampling was for the Discovery group median 55.8 years [IQR 46.9-64] and for the Replication group median 61.8 years [IQR 56.6-71.6] (Mann-Whitney test, p=0.005). In the discovery group 65% of participants were of male sex versus 74% in the replication group (non-significant difference).

### Method details

#### RNA extraction and small RNA sequencing

Blood was centrifuged upon collection and frozen at -80C in our Biobank facility, which ensures no freeze/thaw cycles until measurements took place. Haemolysis was assessed by visual inspection.

Total RNA was purified from serum samples using the miRNeasy Serum/Plasma Kit (Qiagen). RNA was prepared for Illumina sequencing using the QIAseq miRNA Library Kit (Qiagen). Library size selection was done using the beads included in the preparation kit, which generates small RNA sequencing libraries with a broad size profile including tiRNAs. Libraries were subjected to quality control and concentration measurements using the Agilent 2100 Bioanalyzer and qPCR-based library quantification with the KAPA Library Quantification Kit Illumina Platforms (Kapa Biosystems). Raw data from Illumina sequencing was filtered, checked for quality and matched to sncRNAs, including miRNA, small nucleolar RNA (snoRNA), small nuclear RNA (snRNA), and transfer RNA (tRNA) as described below.

#### RNA-seq analysis

The discovery cohort was analysed using the Nextflow based pipeline tsRNAsearch^30^ (last updated version 16 May 2022) with species option set to “human” and nextflow configuration changed to allow increased memory and CPU usage. Nextflow log file is available in the supplemental information. The pipeline performs conventional small RNA-seq data processing step for each input fastq file including adapter removal via TrimGalore^31^ and excluding low quality reads. Reads were aligned to a bespoke database using STAR^32^ and multimapping reads were collapsed at the amino acid levels. Raw counts were generated using featureCounts^33^ and subsequently reformatted to collapse expression at the isodecoder level (or amino acid level for multi-mapped reads) prior to differential expression analysis. In addition, the pipeline uses a custom ncRNA database built based on GtRNAdb and includes 50 nucleotides upstream and downstream of each tRNA. After sequencing the fastq reads have the insert sequence originating from the sequenced RNA, followed by an inner adapter sequence (AACTGTAGGCACCATCAAT), a 12 bp unique molecular index (UMI), and an outer adapter sequence (AGATCGGAAGAGCACACGT): [RNA sequence] AACTGTAGGCACCATCAAT NNNNNNNNNNNN AGATCGGAAGAGCACACGT. The adapter sequence AGATCGGAAGAGC was removed before mapping and reads with minimum length 16 and highest-scoring alignment for each read were retained.

Of note, to independently verify the presence of tsRNAs, a QC plot generated using MultiQC (Figure S3), revealed a peak at 22 bp, consistent with miRNAs, and a secondary broad peak at approximately 30 bp, consistent with expected tiRNA sizes.

We had an average of 10.3M reads after quality checks and adapter trimming (Table S1). Raw sncRNA counts were obtained from tsRNAsearch using DESeq2 method of the pipeline; however, the DESeq2^34^ differential expression analysis was performed independently, as the pipeline does not support paired analysis option. Samples were sequenced in four batches and batch effects were removed using ComBat-seq (Figure S1).^35^ ComBat-seq applies a gene-wise negative binomial regression model to the raw count matrix to estimate and remove batch-associated variance. Patient wise paired analysis was performed using DESeq2 with the following model: ∼ Subject + Timepoint. SncRNAs with low expression were filtered by excluding sncRNAs with less than 10 counts in 20% of the total samples, with 78 sncRNAs retained. DESeq2 works on the raw counts to fit a negative binomial generalized linear model per gene and estimates log2 fold-changes and p-values using the Wald test, with the adjusted p-values calculated using the Benjamini-Hochberg method.^36^ We chose tsRNAs with abs(log2foldchange) greater than 0.50 and adjusted p-value less than 0.05 (Table S2).

#### qPCR analyses of tDR-1:34-Gly-GCC tiRNA

We validated the results from the small RNA-seq analyses using TaqMan-based qPCR analyses of tDR-1:34-Gly-GCC on the samples from the discovery and also for the replication in the independent second cohort. To this end, a custom small RNA Taqman assay (ThermoFisher Scientific) for tDR-1:34-Gly-GCC (5’-GCAUUGGUGGUUCAGUGGUAGAAUUCUCGCCUGC-3’) was designed, optimised, and validated previously.^21^ The assay specifically amplifies tDR-1:34-Gly-GCC. Quantification was performed on a Quantstudio 5 PCR machines (ThermoFisher Scientific). Human tiRNA levels were normalised to *C. elegans* miRNA-39 spike-in, using the 2- Δ ΔCt method. Synthetic *C. elegans* miRNA-39 spike-in was added during RNA purification according to the manufacturer’s instructions (Qiagen serum/plasma miRNeasy kit; 3.5 μl of a 1.6x108 solution added per sample). RNA was eluted in 20 μl water containing 1 μl RNaseOUT ribonuclease inhibitor (Invitrogen) and stored at -80°C.

Per reverse transcription reaction, 2 μl RNA was used, which was performed according to Taqman Small RNA Assay protocol (ThermoFisher). Briefly, 2 μl RNA were added to 3 μl water, then reagents from the TaqMan® MicroRNA Reverse Transcription Kit were added as follows: 0.15 μl dNTPS, 1.5 μl 10 x buffer, 1 μl Reverse Transcriptase, 0.19 μl RNase Inhibitor, and 4.16 μl water. Then 3 μl 5x tDR-1:34-Gly-GCC assay RT primer was added per reaction, total volume 15 μl as per manufacturer’s instructions. An RT minus control was included for each assay. The reactions were amplified according to manufacturer’s protocol, 30 mins at 16°C, 30 mins at 42°C, 5 mins at 85°C and held at 4°C.

One μl of the reverse transcription reaction was used in the qPCR reaction. Briefly, 1 μl cDNA, 7.5 μl TaqMan® Universal PCR Master Mix, No AmpErase® UNG, 0.75 μl 20x tDR-1:34-Gly-GCC qPCR assay and 5.75 μl water were added to a PCR plate, which was sealed and centrifuged prior to loading onto the machine. The plate was incubated at 95°C for 10 mins, then 40 cycles of (94°C for 00:15, 60°C for 01:00) with signal captured at the end of the 60°C step. qPCRs were performed in triplicate and a no-template control was included for each assay. Quality checks on the qPCR process showed a high-performing process, with near all triplicates within the established range of 0.5 Ct values.

#### Angiogenin and neurofilament light ELISA

The concentrations of Angiogenin (ANG) in the serum of ALS patients were evaluated by a human ANG ELISA (DAN00 R&D Systems Inc., Minneapolis, MN) per the manufacturer’s instructions as reported previously.^37^ Samples were stored at -80°C until the assays were performed. The absorbance was measured at 450 nm using a 96-well automatic spectrophotometer (Clariostar, BMG Labtech). Commercially available IVD-labeled ELISAs were used according to the manufacturer's protocol for measuring Neurofilament light (10–7001 CE, Umandiagnostics AB, Umea, Sweden).

### Quantification and statistical analysis

Statistical tests used, definition of center and dispersion are described below. Results are mentioned in the results section and relevant descriptions in the figure legends. The exact value of n, which represents the number of patients, is mentioned in the table and figure legends.

Descriptive data is shown as median and interquartile range for quantitative variables, as most are non-normally distributed, and absolute number with the respective percentages for categorical variables. Differences between cohorts were tested with Mann-Whitney U test for continuous variables and a chi-square test for categorical variables. Given the exploratory nature of this study, no sample size calculations were performed.

Measurements of tDR-1:34-Gly-GCC are expressed as Fold Change (2ˆ- Δ ΔCt). The samples were analysed for each patient individually, comparing timepoint 1 with timepoint 2 and 3, using timepoint 1 as baseline. Survival was measured from disease onset. To assess the relationship between the longitudinal biomarkers and survival, we employed a joint model that combines a longitudinal linear mixed-effects model (with subject ID as random intercept and time since first sampling as a random slope per subject) and a survival model. The within-subject effect of repeated measures was accounted for by the random slope over time, which varied per subject (random slope). Time was also included as a fixed variable, tDR-1:34-Gly-GCC was expressed as raw ΔCt and scaled to 0-1. Associations were based on the current value, not on slope. The Cox survival model was univariable, calculated as survival time since first sample, with death as the endpoint and censoring applied to subjects still alive at the time of dataset closure.

NfL and ANG were log transformed to achieve a normal distribution in the calculations, however for ease of interpretation, figures are in original values. A linear mixed effect model (with subject ID as random intercept and time as a random slope per subject) was used to test the longitudinal effect of clinical parameters on NfL, ANG and tDR-1:34-Gly-GCC, which were corrected for age and sex. Time was included as a fixed variable as categorical timepoints. Analyses were conducted in R version 4.1.2.

## Supplemental information

Document S1. Figures S1–S3

Table S1. Raw RNA-seq data with number of trimmed reads per patient and timepoint

Table S2. Statistical results of all sncRNAs

Data S1. T1 vs. T2 Nextflow log file
