# Supplementary material for: Small RNA sequencing identifies serum tDR-1:34-Gly-GCC tiRNA levels as a biomarker for survival in amyotrophic lateral sclerosis
Source: iScience. 2026 Apr 7;29(5):115636. doi: 10.1016/j.isci.2026.115636 (PMC13127386; doi:10.1016/j.isci.2026.115636)
Supplement: Document S1. Figures S1–S3 [file mmc1.pdf]

iScience, Volume *29*

Supplemental information

Small RNA sequencing identifies serum tDR-1:34-Gly-GCC tiRNA levels as a biomarker for survival in amyotrophic lateral sclerosis

Koen C. Demaegd, Lindy Kool, Sharada Baindoor, Paul D. Donovan, Junyi Su, Henk-Jan Westeneng, Ruben P.A. van Eijk, Grainne Geoghegan, Elena Perez Morrissey, Luise Halang, Koen Poesen, Pegah Masrori, Hesham A.Y. Gibriel, Elisabeth Jirström, Morten T. Venø, Jørgen Kjems, Orla Hardiman, Jan H. Veldink, Kevin Kenna, Leonard H. van den Berg, Philip Van Damme, Jochen H.M. Prehn, and Michael A. van Es

Document S1. Supplemental file containing Figures S1-S3

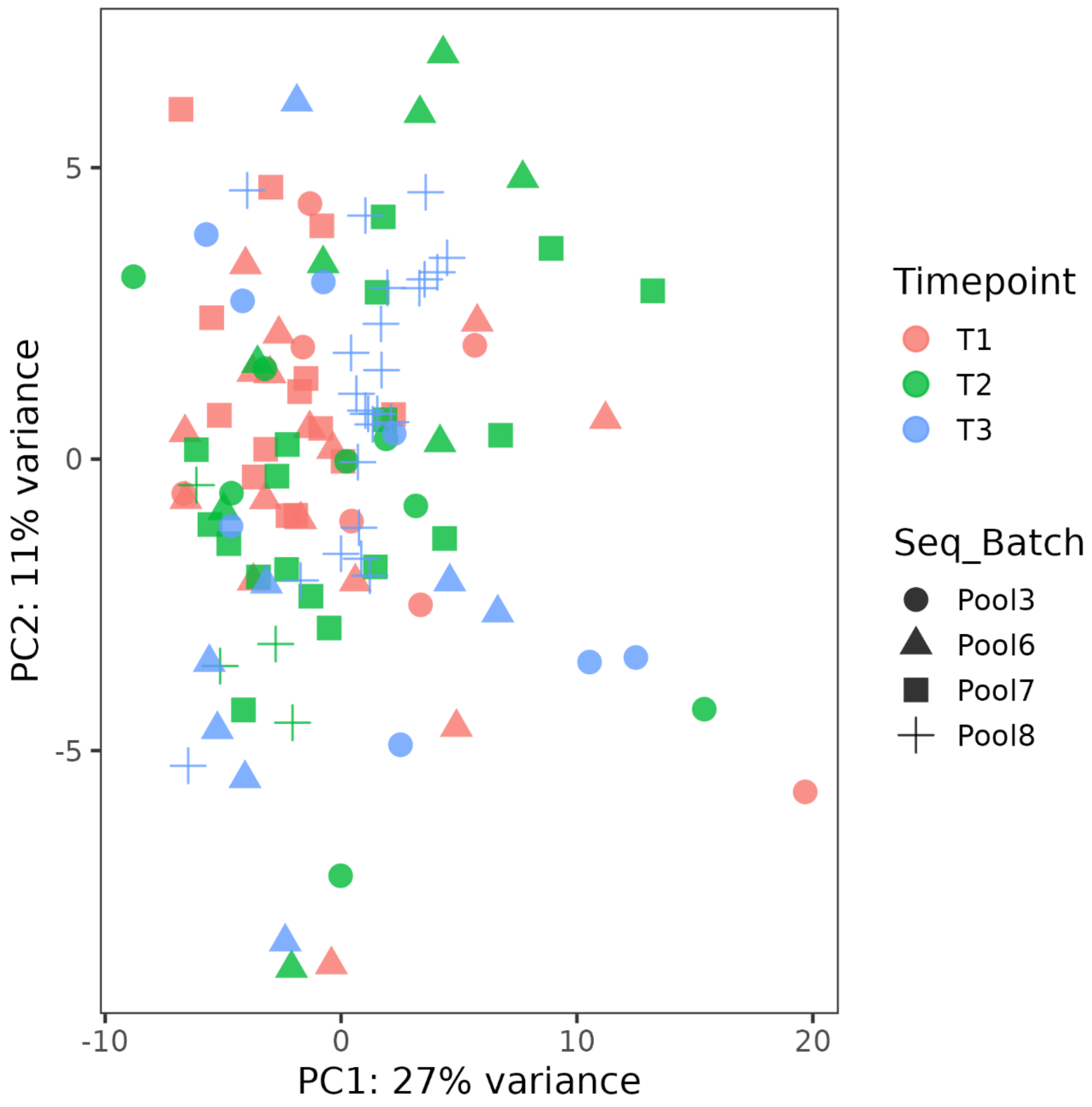

Supplementary figure 1. Assessment of batch effects in RNA-seq data: *Principal component analysis (PCA) plot of variance-stabilised RNA-seq data. Samples are coloured by timepoint (red: T1, green: T2, blue: T3) and shaped by sequencing batch (circle: Pool3, triangle: Pool6, square: Pool7, plus-sign: Pool8). The x-axis (PC1) and y-axis (PC2) represent the first 2 principal components and explains 27% and 11% variance respectively.*

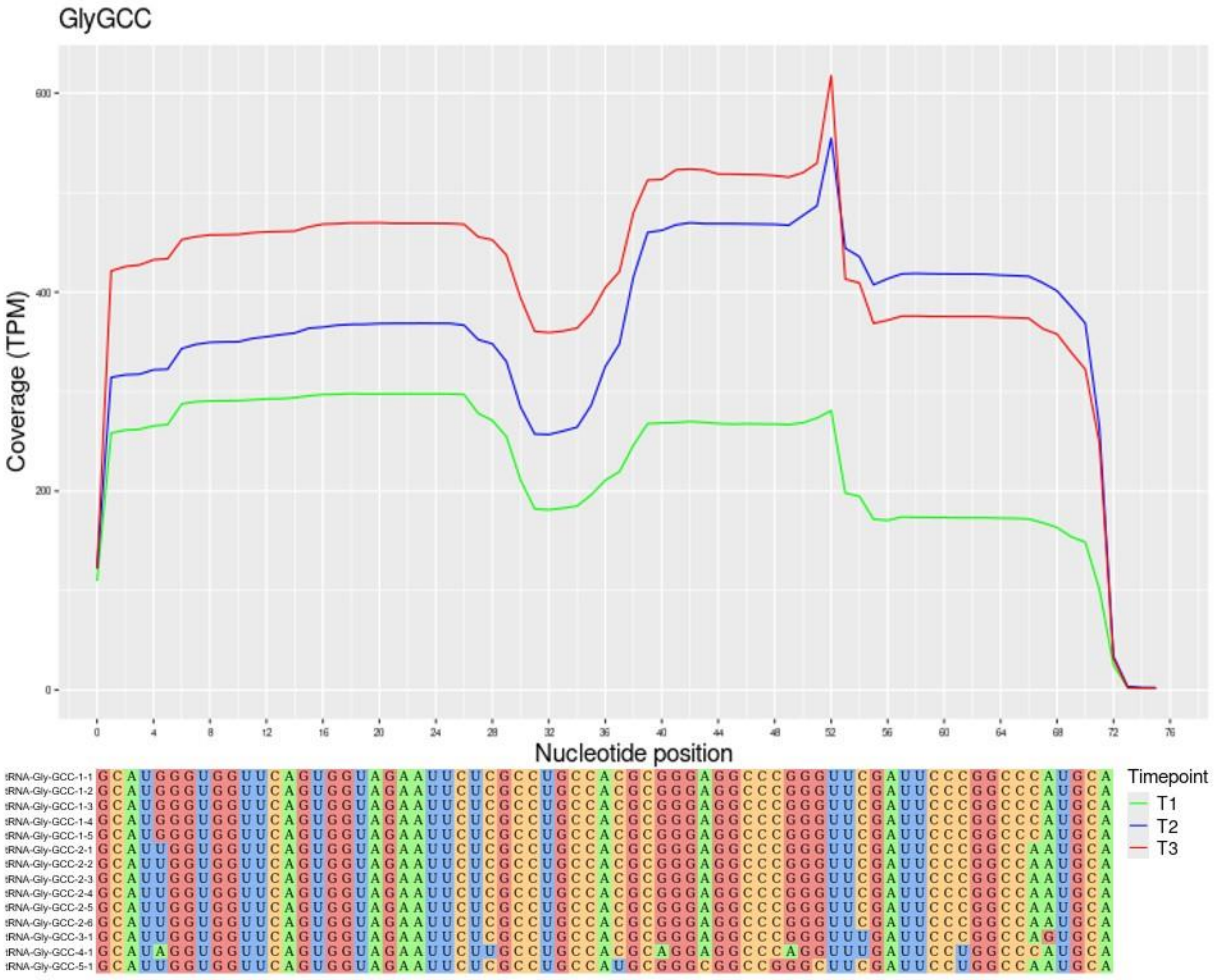

Supplementary figure 2. Coverage plot for GlyGCC expression in RNA-seq data: *Figure shows mean GlyGCC expression colored by timepoints (T1: green, T2: blue, T3: red). X-axis of the line plot shows the nucleotide position and y-axis shows coverage in TPM. Alignment of all GlyGCC sequences included in this study is shown below the coverage plot.*

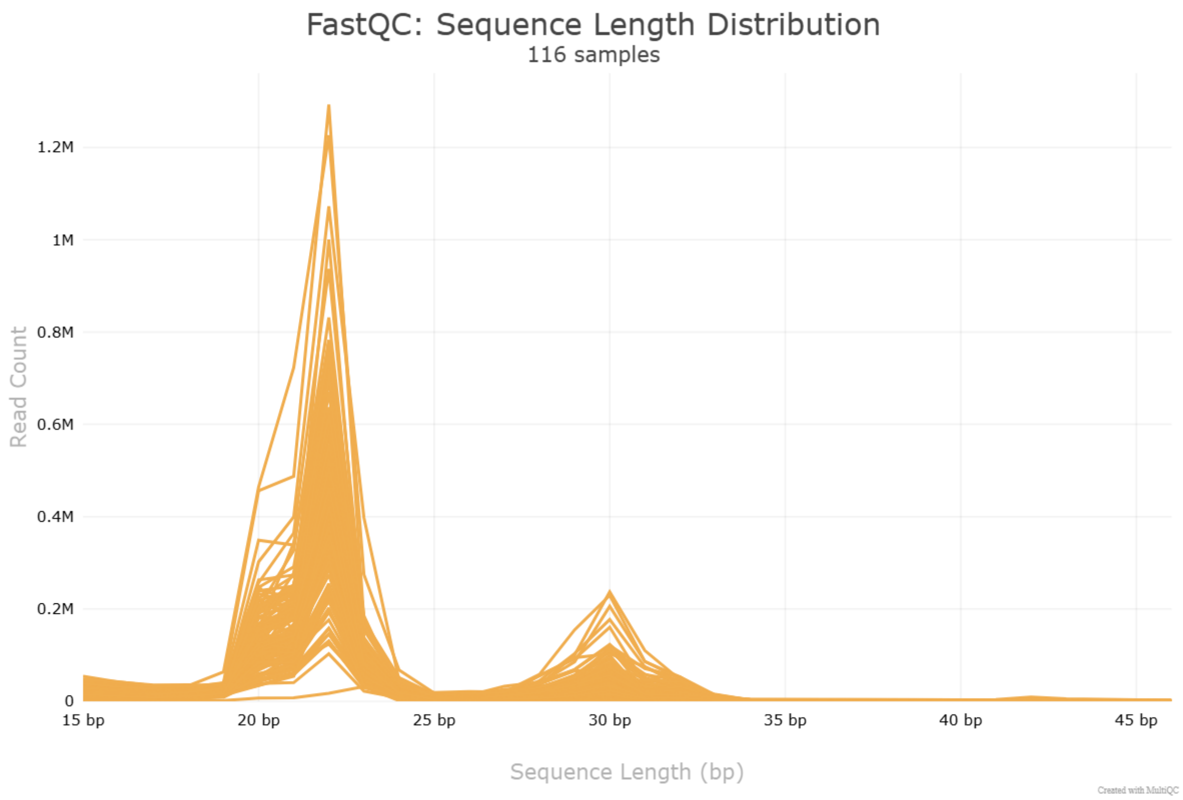

Supplementary figure 3. all fastqc sequence length distribution plot: QC plot showing the insert size after adapter removal for all samples made with multiqc. The main peak is at 22 bp, which is consistent with miRNA, with a secondary broad peak around 30 bp, which fits with tiRNA sizes.
